# Impact of the COVID-19 kindergarten closure on overweight and obesity among 3- to 7-year-old children

**DOI:** 10.1007/s12519-022-00651-0

**Published:** 2022-12-12

**Authors:** Xiang Long, Xing-Ying Li, Hong Jiang, Lian-Di Shen, Li-Feng Zhang, Zheng Pu, Xia Gao, Mu Li

**Affiliations:** 1 Department of Woman and Child Health Care, Jiading Maternal and Child Health Care Hospital, 1216 Gaotai Road, Jiading District, Shanghai 201821, China; 2 School of Public Health, Key Laboratory of Health Technology Assessment (National Health Commission of the People’s Republic of China), Fudan University, Mailbox 175, 138 Yixueyuan Road, Xuhui District, Shanghai 200032, China; 3 Department of Administrative Office, Jiading Maternal and Child Health Care Hospital, 1216 Gaotai Road, Jiading District, Shanghai 201821, China; 4 School of Public Health, The University of Sydney, Sydney, Australia

**Keywords:** Childhood obesity, Childhood overweight, Coronavirus disease 2019 (COVID-19), Kindergarten closure

## Abstract

**Background:**

Knowledge on the impact of the temporary kindergarten closure policy under COVID-19 in 2020 on childhood overweight and obesity is inadequate. We aimed to examine differences in rates of overweight and obesity from 2018 to 2021 among kindergarten children aged 3–7 years.

**Methods:**

Overweight was defined as body mass index (BMI) > 1 standard deviation (SD) for age and sex, and obesity was defined as BMI > 2 SD for age and sex. Generalized linear mixed modeling was used for analysis.

**Results:**

A total of 44,884 children and 71,216 growth data points from all 57 public kindergartens in Jiading District, Shanghai, China were analyzed. The rates of obesity from 2018 to 2021 were 6.9%, 6.6%, 9.5%, and 7.3% in boys and 2.8%, 2.8%, 4.5%, and 3.1% in girls, respectively. The rates of overweight from 2018 to 2021 were 14.3%, 14.3%, 18.2%, and 15.3% in boys and 10.6%, 10.9%, 13.9%, and 11.6% in girls. The rates of obesity and overweight among kindergarten children in 2020 were significantly higher than those in 2018, 2019, and 2021. Compared to 2020, the odds ratios of the obesity rate in 2018, 2019, and 2021 were 0.67 [95% confidence interval (CI) = 0.58–0.77, *P* < 0.001], 0.72 (95% CI = 0.64–0.80, *P* < 0.001) and 0.81 (95% CI = 0.72–0.92, *P* = 0.001), respectively. The odds ratios of the overweight rate in 2018, 2019, and 2021 were 0.75 (95% CI = 0.69–0.82, *P* < 0.001), 0.78 (95% CI = 0.72–0.84, *P* < 0.001), and 0.89 (95% CI = 0.81–0.97, *P* = 0.008), respectively, compared to 2020.

**Conclusions:**

The rates of overweight and obesity significantly increased among kindergarten children in 2020 after the 5-month kindergarten closure. It was critical to provide guidance to caregivers on fostering a healthy lifestyle for children at home under public health emergencies.

**Supplementary Information:**

The online version contains supplementary material available at 10.1007/s12519-022-00651-0.

## Introduction

In recent decades, childhood overweight and obesity (OWO) have become an epidemiological issue of global concern. Childhood OWO is associated with various chronic diseases in adulthood, including hypertension, diabetes, and abnormal cardiac metabolism [1–3]. Furthermore, the prevalence of OWO in children has increased in China, especially in cosmopolitan centers such as Shanghai [4–6]. The prevalence of obesity among children under 7 years of age increased from 0.9% to 4.0% between 1986 and 2016 in Chinese urban areas [4]. In 2016, the overall prevalence of OWO in China was 8.4% and 4.2%, respectively, whereas it was 9.0% and 4.4%, respectively, in Shanghai, higher than the national prevalence [4].

OWO among kindergarten children have been closely associated with unhealthy lifestyles, such as inappropriate diet and lack of physical activities [7, 8]. The school environment, including kindergarten, has been found to be a protective factor for childhood OWO due to the active physical activities and nutritionally balanced meals provided in kindergartens [9–11]. Since the outbreak of coronavirus disease 2019 (COVID-19) [12], many countries and local governments imposed restrictions to control the spread of COVID-19. In 2020, schools, including kindergartens across China, were closed from January [13]. Kindergartens reopened gradually according to COVID-19 situations in different areas. Shanghai's public kindergartens reopened in early June 2020 [14, 15]. Including the winter vacation in January 2020, the duration of stay-at-home for kindergarten children was around five months.

Studies in Italy, the USA, and other countries showed that during the COVID-19 lockdown, food intake and sedentary time increased significantly among children and adolescents, which led to a further increase in body mass index (BMI) or a diminished effect of obesity control [16–21]. Elevated intake of sugary drinks and snacks such as potato chips was common under the COVID-19 lockdown, and there was also a reported decrease in physical activity levels and an increase in sedentary time among children aged 4–18 years, both of which could increase the risk of OWO among children [21–23]. However, very limited studies have used individual-based longitudinal data to examine the impact of COVID-19 kindergarten closure on childhood OWO. Furthermore, most of the existing studies only analyzed data before and immediately after kindergarten closure under the COVID-19 pandemic, and very few of them observed whether the impact of kindergarten closure on childhood OWO would diminish instantly or persist for a period of time after the normal lifestyle resumed. Based on the physical examination database of kindergarten children in Jiading District of Shanghai, China, our study aimed to determine the rates of OWO in 2020 when kindergarten closure occurred due to the COVID-19 pandemic in Jiading District of Shanghai by comparing rates of OWO among kindergarten children in 2020 to those in the pre-COVID-19 years 2018 and 2019, as well as rates of OWO in 2021 after kindergarten reopening. We also observed changes in the rates of childhood OWO in 2021 after kindergarten reopening and lifestyle returning to normal.

## Methods

### Settings

Jiading District is one of the 16 administrative districts in Shanghai, China. It ranks fifth for population size and fourth for GDP among the 16 districts [24, 25]. There were 57 public kindergartens and 18 private kindergartens in Jiading District in 2021. The public kindergartens conducted the physical examination for children within two weeks of reopening in June 2020.

Children’s enrollment numbers in public kindergartens were about four times those in private kindergartens [26]. In addition, the families of children in private kindergartens often had higher mobility, and completed data on children’s physical growth and development were unavailable [27]. For these reasons, our study included children’s growth and development data from all 57 public kindergartens in Jiading District from 2018 to 2021, excluding private kindergartens. This study was approved by the Ethical Committee of Fudan University in Shanghai, China (IRB No. 2020–12-0862).

### Anthropometric measurements

Children in kindergartens in Shanghai usually have physical examinations in May–June each year by experienced child healthcare staff from the district maternal and child healthcare institute and community health centers. They conducted 2018, 2019, and 2021 measurements that were included in this study. However, in 2020, entering kindergartens was strictly restricted due to the COVID-19 prevention and control policy; healthcare teachers in kindergartens conducted children’s physical examinations (anthropometric and eye tests) instead of child healthcare staff. Before the measurements, all kindergarten healthcare teachers in Jiading District received formal training in the district maternal and child healthcare institute to ensure that the same standards and methods would be applied. Weight was measured to 0.01 kg accuracy in light clothing, and height was measured to 1 mm accuracy using a vertical stadiometer. Both weight and height were measured twice, and the average value was taken. BMI was calculated as weight in kilograms divided by height in meters squared (kg/m^2^), and BMI *Z* scores were calculated by BMI means and standard deviations through the World Health Organization (WHO) online anthro survey analyzer for children aged under 60 months and through the R package “anthroplus” for children aged above 60 months. We used the WHO standards, including the child growth standards for 2–5 years old [28] and growth references for 5–19 years old [29], to define childhood OWO. Overweight was defined as BMI for age and gender > 1 standard deviation (SD), and obesity as BMI for age and gender > 2 SD [30].

Data in this study were obtained from all 57 public kindergartens in Jiading District between 2018 and 2021, including one opened in 2019 and three opened in 2021. All data were deidentified. The overall physical examination (anthropometric and eye test) participation rates of all public kindergartens from 2018 to 2021 were 98.3% (20,540/20,882), 99.8% (21,145/21,176), 36.5% (7904/21,660), and 99.6% (21,627/21,717), respectively. The low physical examination participation rate in 2020 was mainly due to the low attendance shortly after the reopening. To ensure that the 2020 data were representative despite the low participation rate, we compared the rates of OWO between the children who returned to kindergarten in June 2020 and children who did not return and found no significant differences in their OWO rates in 2019 (*P* > 0.05).

The raw dataset contained 46,290 children and 72,961 data points, and the dataset was cleaned before the analysis. Data with missing values or duplicated information and outlier BMI values (1% top-end trim) were removed (*n* = 1745). To describe children’s physical growth from 2018 to 2021, we conducted the one-way ANOVA test and Chi-square test, and we included all available physical examinations of the 44,884 children and 71,216 growth and development data points. When conducting generalized linear mixed modeling to compare the rates of OWO over the four years, we used individual-based repeated data. That is, we only included children with at least two years of physical examination records. Therefore, 21,067 children and 47,218 data points were included.

In this study, children were divided into eight age groups according to their age by every six months according to WHO standards for child growth and development [28, 29]. However, in the 6.5-year-old age group, some children failed to enter primary school and stayed in kindergarten because of delayed behavioral development, with a total number of six children. Therefore, in this age group, children’s ages ranged from 78 to 90 months, spanning beyond six months.

### Statistical analysis

For continuous variables, we conducted descriptive analysis, including weight, height, BMI, and mean ± SD. One-way ANOVA and least significance difference (LSD) *t* test were used for comparison of weight, height, and BMI between different years of physical examinations, age, and gender groups. Chi-square test was used to analyze the rates of OWO from 2018 to 2021, including the Mantel‒Haenszel test for trends and Bonferroni's correction for multiple comparisons between four years. The association between experiencing kindergarten closure during the COVID-19 pandemic in 2020 and the rates of OWO among kindergarten children was analyzed using generalized linear mixed modeling (GLMM) [31]. Each kindergarten was introduced into the model as a random effect, while gender, age, and year of physical examination were introduced as fixed effects, with the rate of overweight or obesity as the dependent variable. The significance level was *P* < 0.05, and the data analysis was performed by SPSS 20.0 and R 4.1.3. The figure was made by Prism 9.0.

## Results

### Demographic characteristics

Among the 71,216 growth and development data points of kindergarten children from 2018 to 2021, 28.8% (20,540/71,216) were in 2018, 29.7% (21,145/71,216) in 2019, 11.1% (7904/71,216) in 2020 and 30.4% (21,627/71,216) in 2021. Boys and girls accounted for 53.1% (37,839/71,216) and 46.9% (33,377/71,216), respectively.

### Comparisons of the growth and development of kindergarten children between 2018 and 2021

Supplementary Table 1 provides an overview of the weight, height, and BMI of 3- to 7-year-old boys and girls from 2018 to 2021. For boys, the one-way ANOVA test showed statistically significant differences in weight over the four years (*P* < 0.05), except for the 3.0-year-old age group. There were statistically significant differences in height over four years in the 3.5-, 4.0-, 5.5-, 6.0-, and 6.5-year-old groups (*P* < 0.05). The differences in BMI over four years were statistically significant (*P* < 0.05), except for the 3.0- and 6.5-year-old age groups. The LSD *t* test showed that BMI in all age groups in 2020 was significantly higher than that in 2018 and 2019 (*P* < 0.05), except for the 3.0-year-old age group. The results also revealed that boys' BMI in 2020 was significantly higher than that in 2021 in the 4.0-, 4.5-, 5.5-, and 6.0-year-old age groups (*P* < 0.05). In addition, differences in BMI *Z* scores over four years were statistically significant (*P* < 0.05), except for the 3.0-year-old age group. The LSD *t* test also revealed that BMI *Z* scores were significantly higher in 2020 in all age groups than those in 2018 and 2019 (*P* < 0.05), except for the 3.0-year-old group. In addition, boys’ BMI *Z* scores were significantly higher in 2020 than those in 2021 in the 3.0-, 4.0-, 4.5-, 5.5- and 6.0-year-old age groups (Supplementary Table 1).

For girls, the one-way ANOVA test showed a statistically significant difference in weight over four years in all age groups (*P* < 0.05), except for the 3.0-, 3.5-, and 4.0-year-old groups. There were significant differences for the 3.0-, 5.5-, and 6.0-year-old groups’ height over four years (*P* < 0.05), but not other age groups. The differences in girls’ BMI over four years of all age groups were statistically significant (*P* < 0.05), except for the 3.0- and 3.5-year-old groups. Further LSD *t* test showed that girls’ BMI in 2020 was significantly higher than that in 2018 and 2019 in all age groups (*P* < 0.05) except for the 3.0-year-old group. In addition, girls’ BMI in 2020 was significantly higher than that in 2021 (*P* < 0.05), except for the 3.0-, 5.5-, and 6.0-year-old groups. Girls’ BMI *Z* scores also differed significantly over four years in all groups (*P* < 0.05), except for the 3.0-year-old age group. The LSD *t* test also showed that BMI *Z* scores among girls were significantly higher in 2020 than those in 2018 or 2019 in all age groups (*P* < 0.05), except for the 3.0-year-old group. In addition, girls’ BMI *Z* scores in 2020 were significantly higher than those in 2021 in the 3.5-, 4.5-, 5.0-, and 6.5-year-old groups (*P* < 0.05) (Supplementary Table 1).

### Overweight and obesity rates among kindergarten children from 2018 to 2021

**Fig. 1:**
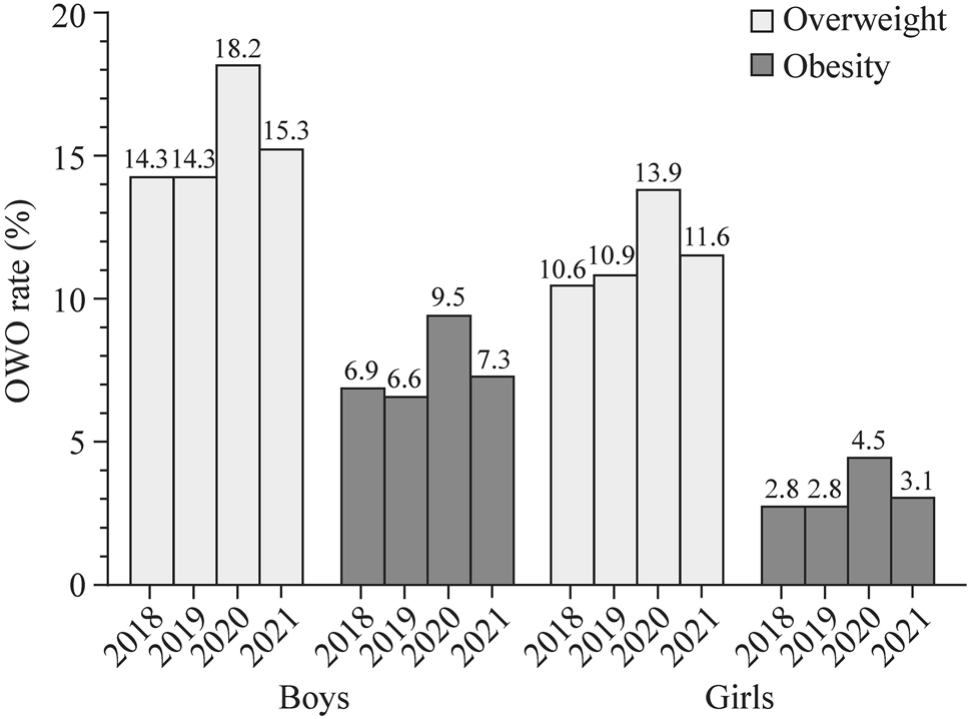
Rates of overweight and obesity (OWO) among kindergarten children from 2018 to 2021 in Jiading District, Shanghai

The rates of OWO among kindergarten boys and girls from 2018 to 2021 are shown in Fig. 1. The overall rate of obesity in boys was 6.9% in 2018, 6.6% in 2019, 9.5% in 2020, and 7.3% in 2021 (Mantel‒Haenszel test for trend *χ*^2^ = 39.506, *P* < 0.001). The rate of overweight in boys was 14.3% in 2018, 14.3% in 2019, 18.2% in 2020, and 15.3% in 2021 (Mantel‒Haenszel test for trend *χ*^2^ = 42.490, *P* < 0.001). The rate of obesity in 2020 was significantly higher than that in 2018, 2019, and 2021 (*P* < 0.05). The rate of overweight in 2020 was also significantly higher than that in 2018, 2019, and 2021 (*P* < 0.001). There were no statistically significant differences in the rates of OWO between 2018, 2019, and 2021 (*P* > 0.05) (Table 1).

**Table 1:** Comparison of overweight and obesity rates among kindergarten children from 2018 to 2021 in Jiading District, Shanghai (*N* = 44,884)

Variables	Boys			Girls		
	Number	Overweight	Obesity	Number	Overweight	Obesity
Total	37,839	5697 (15.1)	2735 (7.2)	33,377	3793 (11.4)	1032 (3.1)
2018	11,284	1617 (14.3)	783 (6.9)	9256	981 (10.6)	256 (2.8)
2019	11,034	1583 (14.3)	728 (6.6)	10,111	1101 (10.9)	283 (2.8)
2020	4227	770 (18.2)	400 (9.5)	3677	511 (13.9)	167 (4.5)
2021	11,294	1727 (15.3)	824 (7.3)	10,377	1200 (11.6)	326 (3.1)
*χ*^2a^		42.490	39.506		31.711	31.883
P^*^		< 0.001	< 0.001		< 0.001	< 0.001

^a^Chi-square test between gender and the rates of overweight and obesity. ^*^P < 0.05 indicates a statistically significant difference

The overall rate of obesity in girls was 2.8% in 2018, 2.8% in 2019, 4.5% in 2020, and 3.1% in 2021 (Mantel‒Haenszel test for trend *χ*^2^ = 31.883, *P* < 0.001). The rate of overweight was 10.6% in 2018, 10.9% in 2019, 13.9% in 2020, and 11.6% in 2021 (Mantel‒Haenszel test for trend *χ*^2^ = 31.711, *P* < 0.001). The rates of OWO in 2020 were significantly higher than those in 2018, 2019, and 2021 (*P* < 0.001). No statistically significant differences were found in the rates of OWO between 2018, 2019, and 2021 (*P* > 0.05) (Table 1).

From 2018 to 2021, there were 21,067 children who had at least two years of physical examinations. We included these children’s data in the GLMM; as a result, 47,218 of 71,216 data points were included in the model. Among the data points included in the GLMM regression, 53.1% (25,093/47,218) were boys and 46.9% (22,125/47,218) were girls.

For all children included in the model, with the rate of obesity as the dependent variable and data from 2020 as the control group, the GLMM regression showed that the rate of obesity was significantly higher in 2020 than that in 2018, 2019, and 2021. Compared to 2020, the odds ratios of the rate of obesity in 2018, 2019, and 2021 were 0.67 [95% confidence interval (CI) = 0.58–0.77, *P* < 0.001], 0.72 (95% CI = 0.64–0.80, *P* < 0.001), and 0.81 (95% CI = 0.72–0.92, *P* = 0.001), respectively, after adjusting for sex and age. Similarly, using the rate of overweight as a dependent variable and data from 2020 as the control group, the rate of overweight was also significantly higher in 2020. The odds ratios of the overweight rate in 2018, 2019, and 2021 were 0.75 (95% CI = 0.69–0.82, *P* < 0.001), 0.78 (95% CI = 0.72–0.84, *P* < 0.001), and 0.89 (95% CI = 0.81–0.97, *P* = 0.008), respectively, compared to 2020, after adjusting for the same factors as in obesity (Table 2).

**Table 2:** Generalized linear mixed model analysis of overweight and obesity in 2020 compared to 2018, 2019 and 2021 (*n* = 21,067)

Variables	Overweight^a^			Obesity^a^		
	OR	95% CI	P^*^	OR	95% CI	P^*^
Total						
2018	0.75	0.69–0.82	< 0.001	0.67	0.58–0.77	< 0.001
2019	0.78	0.72–0.84	< 0.001	0.72	0.64–0.80	< 0.001
2021	0.89	0.81–0.97	0.008	0.81	0.72–0.92	0.001
2020	1.00	–	–	1.00	–	–
Boys						
2018	0.76	0.67–0.85	< 0.001	0.70	0.59–0.82	< 0.001
2019	0.78	0.71–0.87	< 0.001	0.75	0.66–0.86	< 0.001
2021	0.89	0.79–0.99	0.046	0.83	0.71–0.96	0.014
2020	1.00	–	–	1.00	–	–
Girls						
2018	0.75	0.65–0.86	< 0.001	0.62	0.48–0.79	< 0.001
2019	0.77	0.69–0.87	< 0.001	0.65	0.54–0.80	< 0.001
2021	0.89	0.78–1.02	0.092	0.79	0.63–0.99	0.041
2020	1.00	–	–	1.00	–	–

*OR* odds ratio, *CI* confidence interval. ^a^Generalized liner mixed model analysis. ^*^P < 0.05 indicates a statistically significant difference

For boys, with the rate of obesity as the dependent variable and data from 2020 as the control group, the GLMM regression showed that the rate of obesity in boys was significantly higher in 2020. Compared to 2020, the odds ratios of the obesity rate in 2018, 2019, and 2021 were 0.70 (95% CI = 0.59–0.82, *P* < 0.001), 0.75 (95% CI = 0.66–0.86, *P* < 0.001), and 0.83 (95% CI = 0.71–0.96, *P* = 0.014), respectively, after adjusting for age. Similarly, the GLMM regression also showed that the overweight rate of boys was significantly higher in 2020. The odds ratios of the obesity rate in 2018, 2019, and 2021 were 0.76 (95% CI = 0.67–0.85, *P* < 0.001), 0.78 (95% CI = 0.71–0.87, *P* < 0.001), and 0.89 (95% CI = 0.79–0.99, *P* = 0.046), respectively, compared to 2020 after adjusting for age (Table 2).

For girls, with the rate of obesity as the dependent variable and the 2020 data as the control, the regression showed that the rate of obesity was significantly higher in 2020. Compared to 2020, the odds ratios of the obesity rate in 2018, 2019, and 2021 were 0.62 (95% CI = 0.48–0.79, *P* < 0.001), 0.66 (95% CI = 0.54–0.80, *P* < 0.001), and 0.79 (95% CI = 0.63–0.99, *P* = 0.041), respectively, after adjusting for age. Similarly, the GLMM regression revealed that the rate of overweight in 2020 was also significantly higher. The odds ratios of the obesity rate in 2018 and 2019 were 0.75 (95% CI = 0.65–0.86, *P* < 0.001) and 0.77 (95% CI = 0.69–0.87, *P* < 0.001), respectively, compared to 2020. However, no significant differences were shown between the rates of overweight in 2021 and 2020 after adjusting for age (Table 2).

In addition, although the rates of OWO among kindergarten children in 2021 were significantly lower than those in 2020, the GLMM regression showed that they were still higher than those in 2018 and 2019. Compared to 2021, the odds ratios of the obesity rate in 2018 and 2019 were 0.82 (95% CI = 0.71–0.95, *P* = 0.010) and 0.88 (95% CI = 0.79–0.99, *P* = 0.040), respectively. The odds ratios of the overweight rate in 2018 and 2019 compared to 2021 were 0.85 (95% CI = 0.77–0.94, *P* = 0.001) and 0.88 (95% CI = 0.81–0.95, *P* = 0.002), respectively. For both boys and girls, there were no significant differences in the rates of obesity between 2018, 2019, and 2021 (*P* > 0.05). However, the rate of overweight in 2021 in both boys and girls was significantly higher than that in 2018 and 2019. Compared to 2021, the odds ratios of the overweight rate in boys in 2018 and 2019 were 0.85 (95% CI = 0.75–0.97, *P* = 0.013) and 0.88 (95% CI = 0.79–0.98, *P* = 0.021), respectively. For girls, compared to 2021, the odds ratios of their overweight rates in 2018 and 2019 were 0.84 (95% CI = 0.72–0.98, *P* = 0.022) and 0.87 (95% CI = 0.77–0.98, *P* = 0.027), respectively (Table 3).

**Table 3:** Generalized linear mixed model analysis of overweight and obesity in 2021 compared to 2018, 2019 and 2020 (*n* = 21,067)

Variables	Overweight^a^			Obesity^a^		
	OR	95% CI	P^*^	OR	95% CI	P^*^
Total						
2018	0.85	0.77–0.94	0.001	0.82	0.71–0.95	0.010
2019	0.88	0.81–0.95	0.002	0.88	0.79–0.99	0.040
2020	1.13	1.03–1.23	0.008	1.23	1.08–1.40	0.001
2021	1.00	–	–	1.00	–	–
Boys						
2018	0.85	0.75–0.97	0.013	0.85	0.71–1.01	0.059
2019	0.88	0.79–0.98	0.022	0.91	0.79–1.05	0.183
2020	1.12	1.00–1.26	0.046	1.21	1.04–1.41	0.014
2021	1.00	–	–	1.00	–	–
Girls						
2018	0.84	0.72–0.98	0.022	0.79	0.60–1.03	0.079
2019	0.87	0.77–0.98	0.027	0.83	0.67–1.03	0.094
2020	1.12	0.98–1.29	0.092	1.27	1.01–1.59	0.041
2021	1.00	–	–	1.00	–	–

*OR* odds ratio, *CI* confidence interval. ^a^Generalized liner mixed model analysis. ^*^P < 0.05 indicates a statistically significant difference

## Discussion

Our study provided a snapshot of the OWO status of kindergarten children from 2018 to 2021 in China. The results showed that experiencing five months of kindergarten closure during the COVID-19 pandemic in 2020 was associated with a significantly higher risk of childhood OWO. Furthermore, the rates of OWO declined in 2021 but were still higher than in the pre-COVID-19 years.

Children’s lifestyles probably significantly changed during kindergarten closure compared to their usual go-to kindergarten life, which could impact their physical growth and the prevalence of OWO [10, 32, 33]. Physical activities, screen time, and dietary intake have been considered to be closely associated with childhood OWO [33, 34]. A similar study including several cities in China other than Shanghai also pointed out that changes in these lifestyle factors during the COVID-19 kindergarten closure could probably contribute to the increase in OWO prevalence among kindergarten children [35]. The Chinese physical activity guidelines for kindergarten children recommend that children should carry out at least two hours of outdoor activities per day [36]. However, during the COVID-19 pandemic, outdoor activities in public places were discouraged before April 2020 [37]. A Chinese study revealed that in March 2020, kindergarten children spent much less time on physical activities [38]. To improve children’s lifestyles under the COVID-19 public health response policy, the Chinese Early Childhood Development Professional Committee advocated for indoor parent‒child activities [39]. Studies have shown that indoor physical activities have similar effects as outdoor physical activities, such as relieving stress and preventing OWO [40, 41]. However, caregivers might not have adequate knowledge and information about the appropriate indoor activities for children. New social media platforms such as WeChat and Tiktok could be an effective channel for professional institutions such as hospitals and schools to deliver information and encourage children to engage in more home-based exercises [42, 43]. It is also encouraged to design and produce more interactive indoor games and activities for children and caregivers.

During the COVID-19 pandemic, children's total screen time increased significantly in many countries and regions, including Shanghai, China [44–46]. Increasing exposure to electronic devices during kindergarten ages could have adverse effects on the growth and development of children, including increased BMI, adverse impact on social ability, and increased risk of gaming disorders during adolescence [47, 48]. Caregivers should be informed about the possible negative effects of excessive screen time, which could help them be cautious about controlling children's total screen time no longer than the recommended 60 min/day [36].

Research also demonstrated that if children spent more time in front of a screen, they tended to intake more snacks due to distraction and disruption in the formation of food-intake memories [49, 50], resulting in a rise in children's daily energy intake [51]. The increased energy intake might also be linked to people storing calorie-dense food at home during restriction [52]. Furthermore, children's diet patterns could change under kindergarten closure. Kindergartens in China are required to provide food for children to meet the requirements of nutritional balance, including some healthy snacks [53]. Professionals such as kindergarten nutritionists usually develop the menu to ensure that it meets the dietary guidelines [53]. However, it could be difficult for most caregivers to achieve a balanced diet for children at home. Inadequate parenting literacy of caregivers on dietary intake could lead to an unhealthy diet in children. Therefore, it is necessary to provide nutritional guidance for caregivers of children to prepare healthy and regular diets meeting children's nutritional needs at home. For example, kindergartens could provide daily recipes for caregivers to maintain children’s healthy dietary intake [54, 55].

The strength of our study is that we included all 57 Jiading District public kindergarten children’s physical examination data before, during, and after kindergarten closure in 2020 due to the COVID-19 pandemic. The study seized kindergarten closure as an opportunity to observe the impact of lifestyle changes on the OWO rates in Chinese preschool-age children. Although there have been some studies, including Chinese studies, investigating the relationships between the COVID-19 pandemic and physical activity, screen time, and diets [35, 56], few studies have examined the impact of kindergarten or school closures on the prevalence of OWO using large and high-quality school-based anthropometric data and have performed four years of repeated data analysis. The limitation of the study was that private kindergartens were not included, which might lead to selection bias. The OWO rates in private kindergartens were reported to be higher than those in public kindergartens [57]; excluding data from private kindergartens could cause an underestimation of the total rates of OWO among kindergarten children in Jiading District. In addition, our study did not carry out field research to investigate the lifestyles of children, so the findings could only be used to represent the possible impact of policy changes in COVID-19 prevention and control on OWO prevalence in kindergarten children.

In conclusion, kindergarten closure in 2020 was associated with a higher prevalence of childhood OWO than that in 2018 and 2019 before the pandemic. Although the OWO prevalence among kindergarten children fell in 2021 compared with 2020, it was still higher than that before the COVID-19 pandemic. These findings underlined the necessity for healthcare institutions and schools to guide and support caregivers in fostering healthy lifestyles at home, including indoor physical activities, appropriate screen time, and balanced diets, especially in situations of public health emergencies.

## Supplementary Information

Below is the link to the electronic supplementary material.

Supplementary file 1 (DOCX 34 KB)

## Data Availability

Condensed anonymized data are available from the corresponding author on reasonable request.
