# Supplementary material for: Impact of the COVID-19 kindergarten closure on overweight and obesity among 3- to 7-year-old children
Source: World J Pediatr. 2022 Dec 12;19(5):469–77. doi: 10.1007/s12519-022-00651-0 (PMC9742663; doi:10.1007/s12519-022-00651-0)
Supplement: Supplementary file 1 — (DOCX 34 KB) [file 12519_2022_651_MOESM1_ESM.docx]

**Supplementary Table 1.** Weight, height, and BMI of kindergarten children in Jiading District from 2018 to 2021 (*N* = 44,884)

| Age | Year |  | Boys | | | | | Girls | | | | |
| --- | --- | --- | --- | --- | --- | --- | --- | --- | --- | --- | --- | --- |
|  |  | Number | Weight (kg) | Height (cm) | BMI (kg/m^2^) | BMI *Z* scores | Number | | Weight (kg) | Height (cm) | BMI (kg/m^2^) | BMI *Z*  scores |
| 3.0- | Total | 415 | 15.97 ± 2.42 | 101.07 ± 4.16 | 15.57 ± 1.58 | -0.02 ± 1.19 | 247 | | 15.31 ± 1.88 | 99.28 ± 3.86 | 15.49 ± 1.16 | 0.07 ± 0.85 |
|  | 2018 | 200 | 16.13 ± 2.60 | 101.62 ± 4.13 | 15.55 ± 1.66 | -0.05 ± 1.26 | 79 | | 15.42 ± 1.98 | 99.78 ± 4.05 | 15.44 ± 1.26 | 0.01 ± 0.92 |
|  | 2019 | 192 | 15.81 ± 2.28 | 100.52 ± 4.19 | 15.60 ± 1.52 | 0.001 ± 1.14 | 154 | | 15.25 ± 1.82 | 99.04 ± 3.53 | 15.50 ± 1.12 | 0.07 ± 0.82 |
|  | 2020 | 12 | 15.27 ± 1.81 | 101.02 ± 3.69 | 14.94 ± 1.36 | -0.49 ± 1.13 | 3 | | 16.48 ± 1.98 | 104.37 ± 3.38 | 15.10 ± 1.14 | -0.20 ± 0.90 |
|  | 2021 | 11 | 16.51 ± 1.89 | 101.02 ± 3.72 | 16.14 ± 0.91 | 0.50 ± 0.69 | 11 | | 15.11 ± 2.06 | 97.50 ± 5.60 | 15.83 ± 0.97 | 0.55 ± 0.63 |
|  | *F*^a^ |  | 1.080 | 2.307 | 1.142 | 1.382 |  | | 0.564 | 3.240 | 0.488 | 1.434 |
|  | *P*^b^ |  | 0.357 | 0.076 | 0.332 | 0.248 |  | | 0.639 | 0.023 | 0.691 | 0.234 |
| 3.5- | Total | 3736 | 16.92 ± 2.20 | 103.91 ± 3.95 | 15.63 ± 1.36 | 0.15 ± 1.02 | 3280 | | 16.34 ± 2.06 | 102.77 ± 3.94 | 15.43 ± 1.32 | 0.06 ± 0.90 |
|  | 2018 | 1394 | 16.97 ± 2.24 | 104.16 ± 4.01 | 15.60 ± 1.36 | 0.13 ± 1.02 | 1071 | | 16.30 ± 2.08 | 102.84 ± 3.93 | 15.37 ± 1.30 | 0.01 ± 0.88 |
|  | 2019 | 974 | 16.71 ± 2.00 | 103.52 ± 3.88 | 15.56 ± 1.28 | 0.10 ± 0.98 | 904 | | 16.33 ± 2.08 | 102.62 ± 3.97 | 15.47 ± 1.36 | 0.08 ± 0.93 |
|  | 2020 | 318 | 17.28 ± 2.57 | 104.38 ± 4.16 | 15.81 ± 1.64 | 0.28 ± 1.16 | 298 | | 16.60 ± 2.05 | 103.04 ± 4.13 | 15.60 ± 1.18 | 0.18 ± 0.82 |
|  | 2021 | 1050 | 16.93 ± 2.18 | 103.79 ± 3.83 | 15.67 ± 1.35 | 0.18 ± 1.01 | 1007 | | 16.32 ± 2.03 | 102.76 ± 3.86 | 15.42 ± 1.33 | 0.04 ± 0.92 |
|  | *F* |  | 6.194 | 6.789 | 3.220 | 3.315 |  | | 1.795 | 1.042 | 2.504 | 2.873 |
|  | *P* |  | < 0.001 | < 0.001 | 0.022 | 0.019 |  | | 0.146 | 0.373 | 0.057 | 0.035 |
| 4.0- | Total | 6293 | 17.87 ± 2.44 | 106.77 ± 4.16 | 15.62 ± 1.44 | 0.19 ± 1.05 | 5699 | | 17.22 ± 2.31 | 105.61 ± 4.13 | 15.40 ± 1.43 | 0.03 ± 0.94 |
|  | 2018 | 1951 | 17.74 ± 2.38 | 106.59 ± 4.19 | 15.57 ± 1.42 | 0.15 ± 1.03 | 1615 | | 17.18 ± 2.34 | 105.68 ± 4.22 | 15.34 ± 1.40 | -0.01 ± 0.91 |
|  | 2019 | 1747 | 17.75 ± 2.43 | 106.70 ± 4.13 | 15.54 ± 1.41 | 0.13 ± 1.02 | 1725 | | 17.16 ± 2.28 | 105.50 ± 4.02 | 15.38 ± 1.44 | 0.02 ± 0.94 |
|  | 2020 | 565 | 18.08 ± 2.42 | 106.73 ± 4.15 | 15.83 ± 1.46 | 0.34 ± 1.06 | 515 | | 17.28 ± 2.32 | 105.36 ± 4.12 | 15.53 ± 1.46 | 0.12 ± 0.95 |
|  | 2021 | 2030 | 18.03 ± 2.49 | 107.02 ± 4.16 | 15.69 ± 1.48 | 0.24 ± 1.07 | 1844 | | 17.30 ± 2.30 | 105.72 ± 4.14 | 15.43 ± 1.42 | 0.06 ± 0.95 |
|  | *F* |  | 7.561 | 3.749 | 8.376 | 8.696 |  | | 1.367 | 1.688 | 2.948 | 3.215 |
|  | *P* |  | < 0.001 | 0.011 | < 0.001 | < 0.001 |  | | 0.251 | 0.167 | 0.032 | 0.022 |
| 4.5- | Total | 6766 | 19.09 ± 2.83 | 110.29 ± 4.43 | 15.64 ± 1.57 | 0.23 ± 1.09 | 5912 | | 18.40 ± 2.53 | 109.21 ± 4.32 | 15.38 ± 1.44 | 0.01 ± 0.92 |
|  | 2018 | 1744 | 19.09 ± 2.88 | 110.39 ± 4.52 | 15.60 ± 1.56 | 0.20 ± 1.08 | 1483 | | 18.33 ± 2.49 | 109.12 ± 4.46 | 15.35 ± 1.41 | -0.003 ± 0.89 |
|  | 2019 | 2220 | 18.98 ± 2.77 | 110.20 ± 4.45 | 15.57 ± 1.52 | 0.18 ± 1.06 | 1918 | | 18.28 ± 2.51 | 109.12 ± 4.21 | 15.30 ± 1.42 | -0.04 ± 0.91 |
|  | 2020 | 482 | 19.37 ± 2.70 | 110.18 ± 4.25 | 15.91 ± 1.61 | 0.43 ± 1.08 | 449 | | 18.71 ± 2.77 | 109.16 ± 4.43 | 15.64 ± 1.58 | 0.18 ± 0.98 |
|  | 2021 | 2320 | 19.14 ± 2.86 | 110.33 ± 4.38 | 15.67 ± 1.61 | 0.25 ± 1.11 | 2062 | | 18.49 ± 2.52 | 109.38 ± 4.28 | 15.41 ± 1.44 | 0.03 ± 0.93 |
|  | *F* |  | 2.950 | 0.747 | 6.919 | 7.371 |  | | 4.890 | 1.525 | 7.247 | 7.276 |
|  | *P* |  | 0.031 | 0.524 | < 0.001 | < 0.001 |  | | 0.002 | 0.206 | < 0.001 | < 0.001 |
| 5.0- | Total | 6136 | 20.39 ± 3.19 | 113.73 ± 4.64 | 15.70 ± 1.70 | 0.22 ± 1.14 | 5450 | | 19.62 ± 2.98 | 112.64 ± 4.63 | 15.40 ± 1.61 | 0.01 ± 0.98 |
|  | 2018 | 1773 | 20.33 ± 3.23 | 113.90 ± 4.81 | 15.60 ± 1.70 | 0.15 ± 1.15 | 1501 | | 19.64 ± 2.98 | 112.76 ± 4.70 | 15.39 ± 1.60 | -0.0006 ± 0.97 |
|  | 2019 | 2016 | 20.30 ± 3.15 | 113.58 ± 4.59 | 15.68 ± 1.71 | 0.20 ± 1.15 | 1819 | | 19.40 ± 2.87 | 112.51 ± 4.54 | 15.26 ± 1.48 | -0.07 ± 0.94 |
|  | 2020 | 519 | 20.77 ± 3.31 | 114.04 ± 4.68 | 15.90 ± 1.65 | 0.37 ± 1.10 | 505 | | 20.26 ± 3.36 | 112.97 ± 4.61 | 15.81 ± 1.88 | 0.25 ± 1.11 |
|  | 2021 | 1828 | 20.45 ± 3.14 | 113.64 ± 4.51 | 15.78 ± 1.72 | 0.27 ± 1.13 | 1625 | | 19.63 ± 2.95 | 112.57 ± 4.65 | 15.44 ± 1.64 | 0.03 ± 0.99 |
|  | *F* |  | 3.390 | 2.410 | 5.544 | 6.690 |  | | 11.216 | 1.759 | 15.573 | 14.475 |
|  | *P* |  | 0.017 | 0.065 | < 0.001 | < 0.001 |  | | < 0.001 | 0.153 | < 0.001 | < 0.001 |
| 5.5- | Total | 5833 | 21.89 ± 3.67 | 117.14 ± 4.80 | 15.88 ± 1.86 | 0.31 ± 1.22 | 5160 | | 20.85 ± 3.27 | 115.90 ± 4.74 | 15.46 ± 1.72 | 0.02 ± 1.01 |
|  | 2018 | 1868 | 21.67 ± 3.74 | 116.84 ± 5.07 | 15.79 ± 1.87 | 0.24 ± 1.23 | 1589 | | 20.56 ± 3.26 | 115.62 ± 5.13 | 15.32 ± 1.70 | -0.07 ± 1.01 |
|  | 2019 | 1690 | 21.84 ± 3.60 | 117.16 ± 4.67 | 15.83 ± 1.80 | 0.28 ± 1.19 | 1531 | | 20.73 ± 3.12 | 115.90 ± 4.53 | 15.38 ± 1.68 | -0.02 ± 0.99 |
|  | 2020 | 894 | 22.39 ± 3.77 | 117.48 ± 4.78 | 16.15 ± 1.95 | 0.49 ± 1.24 | 715 | | 21.32 ± 3.40 | 116.31 ± 4.62 | 15.70 ± 1.80 | 0.17 ± 1.04 |
|  | 2021 | 1381 | 21.94 ± 3.55 | 117.30 ± 4.58 | 15.88 ± 1.85 | 0.31 ± 1.21 | 1325 | | 21.07 ± 3.35 | 116.04 ± 4.54 | 15.58 ± 1.75 | 0.10 ± 1.01 |
|  | *F* |  | 8.088 | 4.459 | 8.138 | 8.823 |  | | 11.966 | 4.005 | 11.585 | 12.796 |
|  | *P* |  | < 0.001 | 0.004 | < 0.001 | < 0.001 |  | | < 0.001 | 0.007 | < 0.001 | < 0.001 |
| 6.0- | Total | 6032 | 23.44 ± 4.17 | 120.36 ± 4.86 | 16.10 ± 2.09 | 0.37 ± 1.29 | 5301 | | 22.38 ± 3.71 | 119.31 ± 4.79 | 15.66 ± 1.89 | 0.10 ± 1.05 |
|  | 2018 | 1690 | 23.28 ± 4.34 | 120.04 ± 5.06 | 16.06 ± 2.12 | 0.34 ± 1.31 | 1392 | | 22.08 ± 3.82 | 118.94 ± 4.98 | 15.53 ± 1.90 | 0.02 ± 1.08 |
|  | 2019 | 1568 | 23.16 ± 4.02 | 120.44 ± 4.73 | 15.89 ± 1.98 | 0.23 ± 1.26 | 1418 | | 22.24 ± 3.62 | 119.26 ± 4.65 | 15.58 ± 1.86 | 0.05 ± 1.04 |
|  | 2020 | 964 | 23.93 ± 4.04 | 120.45 ± 4.79 | 16.42 ± 2.08 | 0.60 ± 1.25 | 787 | | 22.64 ± 3.65 | 119.47 ± 4.69 | 15.79 ± 1.81 | 0.19 ± 1.02 |
|  | 2021 | 1810 | 23.56 ± 4.18 | 120.55 ± 4.81 | 16.13 ± 2.12 | 0.40 ± 1.29 | 1704 | | 22.63 ± 3.71 | 119.59 ± 4.78 | 15.77 ± 1.92 | 0.16 ± 1.05 |
|  | *F* |  | 8.073 | 3.665 | 13.501 | 16.354 |  | | 7.624 | 5.064 | 6.134 | 7.716 |
|  | *P* |  | < 0.001 | 0.012 | < 0.001 | < 0.001 |  | | < 0.001 | 0.002 | < 0.001 | < 0.001 |
| 6.5- | Total | 2628 | 24.61 ± 4.62 | 122.55 ± 5.01 | 16.29 ± 2.23 | 0.42 ± 1.33 | 2328 | | 23.26 ± 3.93 | 121.45 ± 5.00 | 15.70 ± 1.89 | 0.08 ± 1.02 |
|  | 2018 | 664 | 24.28 ± 4.72 | 121.99 ± 5.23 | 16.22 ± 2.33 | 0.36 ± 1.37 | 526 | | 23.02 ± 3.70 | 121.24 ± 5.10 | 15.60 ± 1.75 | 0.04 ± 0.96 |
|  | 2019 | 627 | 24.35 ± 4.77 | 122.40 ± 4.90 | 16.15 ± 2.26 | 0.33 ± 1.36 | 642 | | 23.02 ± 3.94 | 121.41 ± 4.94 | 15.54 ± 1.96 | -0.15 ± 1.08 |
|  | 2020 | 473 | 24.94 ± 4.32 | 122.66 ± 4.98 | 16.49 ± 2.08 | 0.57 ± 1.24 | 405 | | 23.85 ± 4.19 | 121.62 ± 5.07 | 16.04 ± 1.97 | 0.28 ± 1.00 |
|  | 2021 | 864 | 24.87 ± 4.57 | 123.03 ± 4.88 | 16.34 ± 2.19 | 0.47 ± 1.30 | 755 | | 23.31 ± 3.92 | 121.54 ± 4.95 | 15.70 ± 1.85 | 0.10 ± 1.01 |
|  | *F* |  | 3.502 | 5.733 | 2.469 | 3.856 |  | | 4.525 | 0.551 | 6.401 | 7.580 |
|  | *P* |  | 0.015 | < 0.001 | 0.060 | 0.009 |  | | 0.004 | 0.647 | < 0.001 | < 0.001 |

*BMI* body mass index. One-way ANOVA test among weight, height and BMI from 2018 to 2021 in each age group; b*P* < 0.05 indicates a statistically significant difference.
